# Advances in All-Inorganic Perovskite Nanocrystal-Based White Light Emitting Devices

**DOI:** 10.1021/acsomega.3c00188

**Published:** 2023-05-11

**Authors:** Tajamul A. Wani, Javad Shamsi, Xinyu Bai, Neha Arora, M. Ibrahim Dar

**Affiliations:** † Department of Materials Science and Engineering, Indian Institute of Technology Delhi, New Delhi 110016, India; # Cavendish Laboratory, Department of Physics, University of Cambridge, Cambridge CB3 0HE, United Kingdom; ° Department of Chemistry, University College London, London WC1H 0AJ, United Kingdom

## Abstract

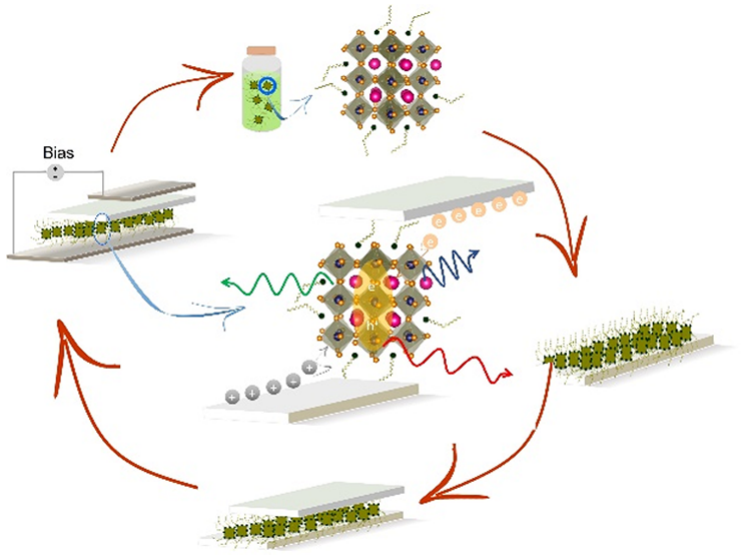

Metal halide perovskites (MHPs) are exceptional semiconductors best known for their intriguing properties, such as high absorption coefficients, tunable bandgaps, excellent charge transport, and high luminescence yields. Among various MHPs, all-inorganic perovskites exhibit benefits over hybrid compositions. Notably, critical properties, including chemical and structural stability, could be improved by employing organic-cation-free MHPs in optoelectronic devices such as solar cells and light-emitting devices (LEDs). Due to their enticing features, including spectral tunability over the entire visible spectrum with high color purity, all-inorganic perovskites have become a focus of intense research for LEDs. This Review explores and discusses the application of all-inorganic CsPbX_3_ nanocrystals (NCs) in developing blue and white LEDs. We discuss the challenges perovskite-based LEDs (PLEDs) face and the potential strategies adopted to establish state-of-the-art synthetic routes to obtain rational control over dimensions and shape symmetry without compromising the optoelectronic properties. Finally, we emphasize the significance of matching the driving currents of different LED chips and balancing the aging and temperature of individual chips to realize efficient, uniform, and stable white electroluminescence.

## Introduction

Metal halide perovskites (MHPs) possess an ABX_3_ (X = Cl, Br, I) type structure in which a divalent B-site cation is coordinated to six halide ions, forming a regular octahedral structure, and a monovalent A-site cation is coordinated to 12 halide ions to form cuboctahedral structures. The three-dimensional ABX_3_ framework consists of corner-sharing [BX_6_]^4–^ octahedra with monovalent cations, such as methylammonium CH_3_NH_3_^+^ (MA), formamidinium HC(NH_2_)_2_^+^ (FA), or Cs^+^, occupying the voids. These voids are created by four neighboring [BX_6_]^4–^ octahedra, resulting in cubic or pseudocubic structures. The metal halide framework contributes electronic states to the valence and conduction bands, whereas the A-site cations like MA^+^ enable the formation of a 3D perovskite crystal structure.^1a,1b^ Although the monovalent A-site cations do not directly contribute any electronic states to the valence or conduction band, they substantially influence the bandgap and other optoelectronic properties by manipulating the bond length and bond angle. A relatively smaller B-site cation (Sn^2+^ vs Pb^2+^) decreases the bandgap, whereas a smaller halide (Cl^–^ vs I^–^) increases the bandgap of ABX_3_.

Although tremendous improvement has been realized in the performance of both solar cells and light-emitting devices (LEDs), relatively modest long-term operational stability has hindered the commercialization of perovskite devices. Fundamentally, the stability issues arising from the degradation of the active layer could be either due to the chemical affinity of the A-site cation or due to the poor stability of the [PbX_6_]^4–^ inorganic framework under illumination or applied bias.^1c,1d^ Among the commonly used A-site cations, Cs offers great chemical stability, primarily due to the symmetric charge distribution or absence of any permanent dipole moment.^1e^ This motivates us to employ all-inorganic perovskite layers for application in solar cells and LEDs. In contrast to efficient light harnessing, efficient light emission demands a narrow line width with great tunability, which could be achieved by a uniform size distribution and reducing the dimensions of all-inorganic perovskite nanocrystals (NCs), respectively. In particular, the peculiar optical properties like narrowband emission with a full-width at half-maximum (fwhm) below 20 nm, wide wavelength tunability (400−700 nm), high photoluminescence quantum yield (PLQY),^2^ reasonable carrier diffusion wavelengths,^3^ low nonradiative recombination rates, high external quantum efficiency, and high current efficiency^4a^ make them potential candidates for application in LEDs.

To realize a 100% PLQY, which is a ratio of the number of photons emitted to the number of photons absorbed, parasitic losses associated with nonradiative recombinations need to be eliminated. Intrinsically, MHPs are defect-tolerant, and remarkable quantum efficiencies can be obtained from a wide range of samples, including colloidal solutions, thin films, nanoparticles, and large crystals obtained through facile bottom-up solution-based approaches. These high-PLQY-yielding samples enabled the fabrication of perovskite-based LEDs (PLEDs) displaying remarkable external quantum efficiencies (EQEs). The EQE, which is a ratio of the number of photons emitted to the number of electrons injected into the devices, cannot be as high as the PLQY, as charge injection layers and interfacial processes play a vital role in determining the overall performance of both solar cells and LEDs.^4b,4c^ In addition to the PLQY, also known as the internal quantum efficiency, the EQE depends on the injection efficiency (the proportion of total charges injected into the emitter layer) and collection efficiency (the proportion of photons generated in the emitter layer that escape from the LED). Therefore, the optimization of interfaces, which can directly improve injection and collection efficiencies, has been pursued intensively to obtain highly efficient and operationally stable devices.^4d^

The basic working principle remains the same for all PLEDs. In terms of performance, tremendous progress has been made for red and green PLEDs, with EQEs reaching 23% and 28%, respectively.^5a,5b^ In contrast, poor stability due to the high applied potential to achieve peak performances and the relatively modest performance of blue emitters have substantially hindered the overall development of white LEDs.^5a,5b^ Therefore, to accelerate the applications of white perovskite LEDs, the stability and efficiency of the blue emitter need to be improved. The PLED community is striving to design and fabricate stable and efficient tunable LEDs, and arguably all-inorganic emitters hold immense potential.

Reasonable development in all-inorganic perovskite NCs to generate emissions covering multiple wavelengths can lead toward generating RGB (red, green, and blue) for white light emission. The applications of all inorganic perovskite NCs in lasers, solar cells, photodetectors, field effect transistors (FETs), and photocatalysis, among others, have been thoroughly discussed elsewhere.^5c,5d^ In this Review, we focus on the emergence of white LEDs based on all-inorganic perovskite NCs. We first discuss the challenges faced and strategies adopted while establishing state-of-the-art synthetic routes to obtain precise control over size and morphology without compromising on critical optoelectronic properties. This follows the application of CsPbX_3_ NCs used in wide bandgap semiconductors for blue LEDs, with their extension into white-light emission. Finally, we put forth the challenges that PLEDs are facing and the potential strategies that could help mitigate the issues, for example, mitigating the toxicity of Pb by employing perovskite-inspired Cu-based halide NCs. In summary, a strong emphasis is given to facilitating efficient carrier transport and hole injection to realize efficient and stable white electroluminescence.

## Synthesis of Blue-Emitting All-Inorganic Perovskite Nanocrystals

**Figure 1:**
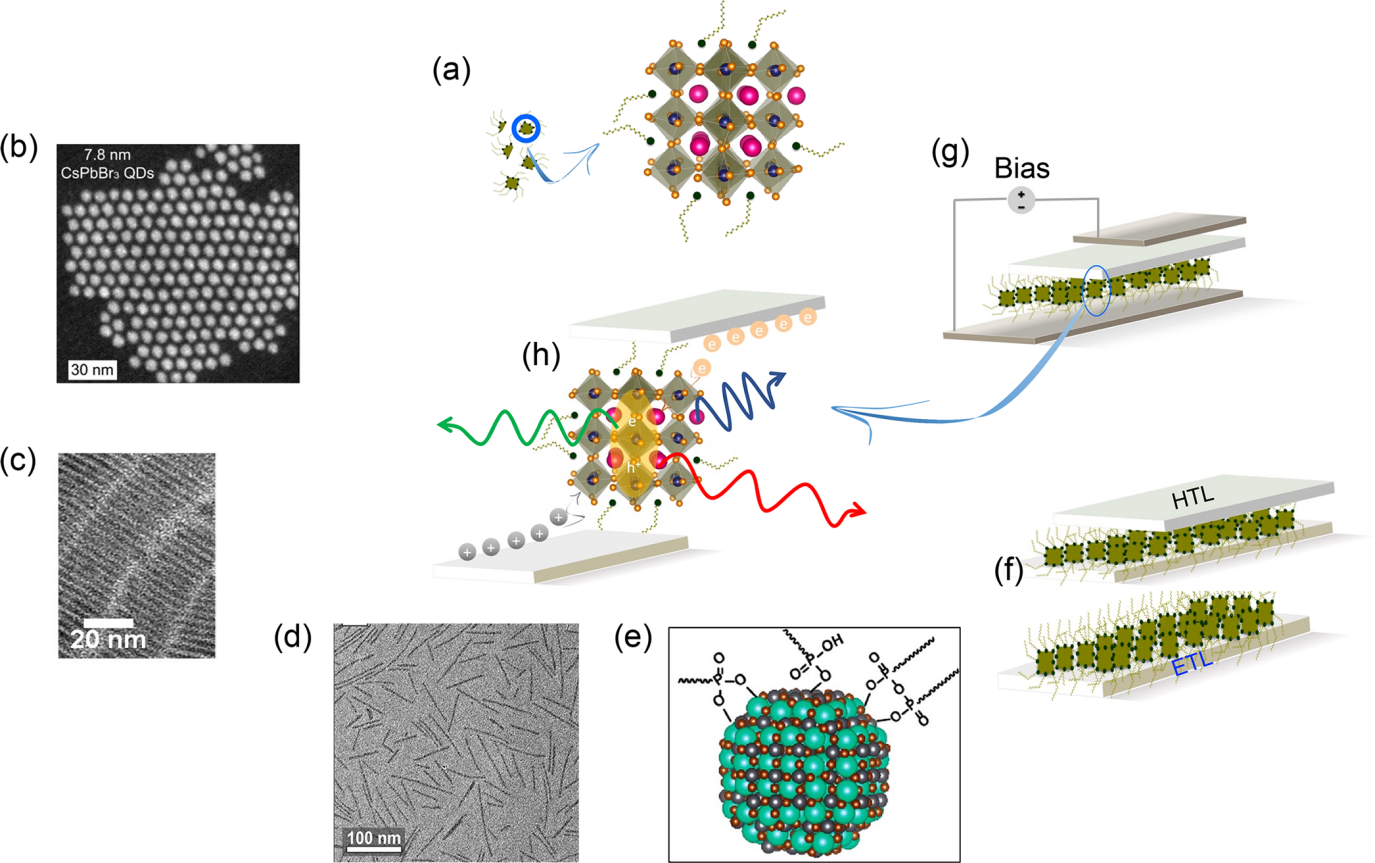
(a) Perovskite NC entities in the stock solution (left) and the 3D arrangement of perovskite structures (right). Pink balls represent the monovalent cation Cs^+^, orange balls indicate the face-centered halide ions X^–^, and blue balls at the center of octahedra are divalent Pb^2+^ cations. (b) STEM image of monodisperse CsPbBr_3_ QDs. Adapted with permission from ref (6c). Copyright 2022 Science. (c) TEM image of anisotropic CsPbBr_3_ nanoplatelets. Adapted from ref (8a). Copyright 2020 American Chemical Society. (d) HRTEM image of flat flying 2D NRs. Adapted with permission from ref (8b). Copyright 2022 American Chemical Society. (e) Schematic illustration of OLPA-based CsPbBr_3_ nanocrystals passivated by hydrogen phosphonates, phosphonic acid anhydrides, and phosphonate species. Adapted with permission from ref (9d). Copyright 2020 American Chemical Society. (f) Perovskite deposition on ETL and HTL deposition on top of the perovskite layer. (g) Device operation of a fully fabricated LED upon applying bias. (h) Electron–hole recombination process inside the perovskite layer.

High-quality MHP NCs in the form of colloidal semiconductor inks (Figure 1a) are synthesized using solution-based approaches. Like other NC systems, a diverse range of methods, such as ligand-assisted reprecipitation (LARP), hot injection (HI), solvothermal, microwave-assisted (MWA), ultrasonication, mechanochemical synthesis, printing, template-assisted, anion exchange, etc., have been adopted to obtain all inorganic NCs.

LARP is a room-temperature process in which the precursor salts and ligands dissolved in a polar solvent, e.g., dimethylformamide (DMF), are injected into the nonpolar solvents (hexane, toluene, etc.).^5e^ HI relatively occurs at higher temperatures >140 °C; the precursor solution containing the Cs^+^ ion is injected into a hot solution containing ligands and lead salts.^5f^ MWA enables homogeneous heating and prevents the hassle of preparing two solutions, like in HI. All the constituents are added to the microwave quartz tube, and then the tube is placed inside the microwave reactor at a specific temperature >150 °C.^5g^ The solvothermal method involves placing precursors, solvents, and ligands inside the autoclave for a specific duration at a specific temperature.^5h^ Ultrasonication is another approach in which NCs are directly prepared from their precursors. All the constituents—a mixture of Cs/Pb salts and ligands—are directly dissolved in nonpolar solvents.^5i^ In mechanochemical synthesis, the Cs and Pb salts are mixed and milled by high-speed balls at ambient temperature.^5j^ A few other methods, such as anion exchange and postsynthetic treatments, are discussed ahead.

Extensive research has been carried out to optimize the synthesis of perovskite nanocrystals over the past few years, and numerous research articles have been published. However, due to their ionic nature, MHP NCs form instantaneously. Hence, exploring their growth kinetics has been challenging. By solely relying on the thermodynamic equilibrium of the Br^–^ content between the solution medium and the quantum dot (QD) lattice, Dong et al. achieved size tuning in perovskite QDs. Increasing the Br^–^ content decreased the QD size and shifted the PL from 498 to 467 nm.^6a^ Furthermore, pure blue-emitting CsPbBr_3_ QDs with an emission wavelength around 460 nm, PLQY as high as 98%, a high exciton binding energy (*E*_b_ = 301.6 meV), and impressive stability was developed by exploring quantum confinement. Ultrasmall NCs were obtained by pouring liquid nitrogen (LN) into the solution containing hydrogen bromide (HBr) and toluene. The authors demonstrated that lowering the synthesis temperature is mandatory to impede the ultrafast nucleation and growth of CsPbBr_3_ NCs. The resulting ultrafine NCs show a value of *E*_b_ 3× higher than that of the untreated solution, presumably due to the lattice contraction due to increased Cs–Br and Pb–Br interactions. Such a high binding energy indicates that PL emission occurs through exciton recombination. The high PLQY again demonstrates the ability of LN to passivate nonradiative recombination pathways effectively. An increase in stability was verified by monitoring the PL peak position, which remained unchanged for 60 days.^6b^ To achieve precise control over the dispersion and dimensions of nanocrystals, it is important to separate the nucleation process from the growth. Recently, Akkerman et al. proposed a new synthetic route to separate the kinetics of nucleation and growth by tuning the equilibrium between the precursor and solute, resulting in monodisperse QDs ranging from 3 to 13 nm (Figure 1b).^6c^ Although this study has been a breakthrough in the field, it would be interesting to realize asymmetric shapes like nanoplatelets (NPLs) and nanowires (NWs) using this approach.

Interestingly, almost all morphologies ranging from 0D (QDs), 1D (NWs), and 2D (nanosheets - NSs) to quasi-2D nanostructures of MHPs have been synthesized using standard labile ligands (primary amines).^7a^ The resulting nanostructures are relatively less stable compared to their 3D counterparts. The labile primary amine ligands generally allow coalescence. Furthermore, the optical properties of the nanostructures are strongly influenced by the large density of surface defects due to the large surface-to-volume ratio, which increases in low-dimensionality systems and thus impedes their further exploration for investigations and applications. Liang et al. synthesized blue-emitting CsPbBr_3_ NCs in various shapes (QDs, stacking 2D NPLs, and flat-lying 2D NSs), and the shape tuning was achieved by varying the ratio of oleic acid (OA) and oleylamine (OAm). The different morphologies displayed emissions ranging from deep blue to sky blue.^7b^ The parameters related to these NCs, such as PLQY, PL, size, and fwhm, are provided in Table 1. Postsynthesis treatment is a promising strategy to passivate surface defects in nanomaterials with reduced dimensionality. When strongly quantum-confined 2D CsPbBr_3_ NPLs emitting around 464 nm were treated with a PbBr_2_–ligand solution, the PLQY increased to 73%, presumably enabled by the passivation of Br^–^ and Pb^–^ vacancies.^7c^

**Table 1:** Summary of Synthetic Routes, Sizes, Spectral Features, and Stability of All-Inorganic Perovskite Nanostructures

perovskite system	synthesis route	size (nm)	PL (nm)	fwhm (nm)	PLQY (%)	stability in air	ref
CsPbBr_3_ QDs	HI–thermodynamic equilibrium	3.7–6.2	467–498	94–142 meV	80–95		(6a)
CsPbBr_3_ QDs	low-temperature thermodynamic suppression	3.0	460	12	98	60 days	(6b)
CsPbBr_3_ QDs (0D)	HI	2.4	453	22	50.41		(7b)
CsPbBr_3_ QDs (1D)		3.6	472		35.70		
CsPbBr_3_ NPLs		2.3	449		54		
CsPbBr_3_ NPLs	RT synthesis and postsynthetic treatment	1.2	464	11	73		(7c)
CsPbBr_3_ cuboid NCs	spontaneous self-assembly	50 × 50 × 20	480	21	91	45 days	(7d)
CsPbBr_3_ NPLs	RT synthesis and in situ cross-linking passivation	18	466	14	100	30 days	(7e)
CsPbBr_3_ NPLs	colloidal synthesis	2.4	450	15	40	1 month	(8a)
CsPbBr_3_ NRs	HI	3.4	471		60		(8b)
CsPbX_3_ NRs	kinetic control	3.8		21			(8c)
CsPbBr_3_ 12 faceted dodechahedron NCs	HI	30			100		(9a)
CsPbBr_3_ truncated octahedron NCs	HI	7–17	498–518	83–125 meV	72–97		(9c)
CsPbBr_3_ truncated octahedron NCs	HI	5–9.2	491–509		81–91		(9d)
CsPbBr_3_ nanoclusters	HI	2	410			2 weeks	(9b)
CsPbBr_*x*_Cl_3–*x*_ NCs	LARP	4–10	406–488	12–18	10–89		(10a)
CsPbBr_3_:Sb^3+^ NCs	LARP	2.2–2.9	461	14	73.8	20 days	(10c)
CsPb(X)_3_:Cu	HI		453	23	80	30 days	(10d)
CsPbCl_3_:La^3+^/F^–^	HI	8.7–9.3	411		36.5		(10e)

A synthetic strategy to eliminate nonradiative recombination pathways involves the minimization of defects. In this direction, a superlattice or multiple quantum wells were developed by spontaneously self-assembling CsPbBr_3_ NPLs into cuboid NCs. This effectively reduced trap-state densities, eventually leading to higher values of PLQY (91%) at 480 nm and a reduction in energetic disorder (Urbach energy of 40 meV).^7d^ To further boost the quantum efficiency, traps within the CsPbBr_3_ NPLs and on the surface require passivation. By employing an in situ cross-linking strategy using (3-aminopropyl)triethoxysilane (APTES) as a cross-linking agent, both surface and deep traps were eliminated, which led to the realization of 100% PLQY and ultrapure emission at 466 nm with fwhm of ∼14 nm.^7e^ Besides the quantum efficiency, it is equally essential to improve stability. Emissive and stable quantum-confined CsPbBr_3_ NPLs were synthesized by introducing hexylphosphonic acid (HPA) in the precursor solution. The strong binding affinity of HPA with the surface of NPLs prevented coalescence in solution and solid-state film samples even upon the injection of a high charge carrier density. The resulting NPLs (Figure 1c) exhibit an excitonic absorption band at 445 nm and blue emission at 450 nm.^8a^ Considerable efforts were devoted to growing nanorods (NRs) to further improve other properties. For example, 1D CsPbBr_3_ NRs were synthesized using a mixture of alkyl amine and carboxylic acids as ligands (Figure 1d). The length of the NRs was controlled by varying the amount of antisolvent at the purification stage. The stability of the NRs toward moisture and polar solvents was improved by using amino-terminated poly(styrene)-*block*-poly(1,4-isoprene).^8b^ In another work, the injection rate of precursor solutions was exploited to obtain quantum-confined 1D CsPbX_3_ NRs. The evolution of NRs with the reaction time was studied by monitoring the PL and absorption features.^8c^ The emission stability was improved by functionalizing 12-faceted dodecahedrons with tertiary ammonium ions ligands like the *N*,*N*-diphenyl oleylammonium ion (DPOA). DPOA-treated CsPbBr_3_ NCs showed a minimal drop in PLQY even after successive stages of precipitations, redispersion, and Br additions.^9a^ Quantum-confined CsPbBr_2.3_ nanoclusters (NCLs) with a distorted orthorhombic structure and stability for up to 2 weeks were prepared using OAm and benzoic acid. These NCLs are disc-shaped with hexagonal short-range order and lamellar long-range order.^9b^

Besides amine and carboxylate anchoring groups, relatively strong binding alkyl phosphonic acids ligands have been explored.^9c,9d^ Truncated octahedron-shaped NCs were synthesized by employing alkyl phosphonic acids as the only ligands during synthesis. The NCs exhibit PLQYs of 92.9%, 95.3%, and 96.8% with HPA:ODPA (octadecylphosphonic acid), tetradecylphosphonic acid (TDPA), and TDPA:ODPA, respectively. Similarly, oleylphosphonic acid (OLPA) was used to grow stable and size-tunable CsPbBr_3_ QDs with truncated octahedron shapes. Size tunability between 5.0 to 9.2 nm was achieved by varying the reaction time during synthesis from 45 to 600 s at 100 °C. OLPA-treated NCs offer higher stability in apolar solvents. The truncated octahedron shape of alkyl phosphonic acids and OLPA-treated NCs is due to the strong binding affinity of phosphonate groups toward (001) and (110) Pb^2+^-terminated facets (Figure 1e).

Anion exchange has also been exploited to tune the emission toward the blue region in perovskite NCs. The halide exchange occurs relatively quickly in perovskite NCs without inducing structural changes. With this possibility, CsPbBr_3_ QDs were transformed into monodisperse CsPbCl_3_ and CsPb(Cl/Br)_3_ at room temperature by in situ anion exchange with ZnCl_2_, preserving both the shape and size of the parent CsPbBr_3_ QD, which is reflected in the identical excitonic absorption transitions in both parent and final QDs.^9e^

Besides confining the size or reducing dimensions, compositional engineering has been explored to tune and tailor the emission features. The CsPbBr_*x*_Cl_3–*x*_ mixed halide perovskite NCs exhibited emission from blue to sky blue when the Br concentration was increased.^10a^ Furthermore, by employing tetrabutylammonium *p*-toluenesulfonate (TBSA) during the purification process, the mixed halides CsPbBr_*x*_Cl_3–*x*_ were manipulated to tune the spectra toward the blue region of the spectrum. TBSA promotes the exchange of Br with Cl anions in CsPbBr_1.5_Cl_1.5_ NCs, and an increase in the concentration of TBSA causes more Br to be replaced with Cl, which shifts the emission from 457 to 409 nm.^10b^ Besides halides, cationic dopants have been investigated to tailor the growth and emission properties of perovskite nanocrystals. To limit the growth of perovskite NCs, CsPbBr_3_ NCs were doped with Sb^3+^, and very small blue-emitting CsPbBr_3_ NCs with emission around 460 nm (fwhm ∼14 nm) were obtained.^10c^ By adopting a similar approach, Cu^2+^-incorporated CsPbX_3_ (X = Br, Br/Cl), i.e., CsPb_1–*x*_Cu_*x*_X_3_ NCs revealed an improvement in thermal stability alongside inreased emission yields.^10d^ The introduction of relatively smaller cations (Sb^3+^, Cu^2+^, etc.,) as compared to Pb^2+^ into perovskite NCs induces lattice contraction and increases the dopant–halide interaction. Improving the short-range order of the lattice consequently enhances the lattice formation energy and also improves the stability. Blue–violet-emitting CsPbCl_3_ NCs doped with Li^3+^ and F^–^ enabled the modification of Cl vacancies, which eventually enhanced the recombination rate.^10e^

The colloidal NCs are transferred to a substrate containing a charge injection layer (electron or hole conductor) (Figure 1f) using spin or spray coating methods or manufacturing techniques such as inkjet printing, among others. Finally, the device is completed by depositing a back-contact layer (Figure 1g). Under operational conditions, the injected carriers, i.e., electrons and holes, recombine inside the perovskite material sandwiched between electron and hole conductors (Figure 1h). Bright emission is generally realized when the injected carriers recombine radiatively, which requires the minimization of nonradiative recombination centers (traps) both within the perovskite NCs and at the interfaces. The strategies adopted for the passivation of nonradiative recombination centers in perovskite solar cells could be extended to LEDs as well.^4d^

## All-Inorganic Perovskite Blue LEDs

**Figure 2:**
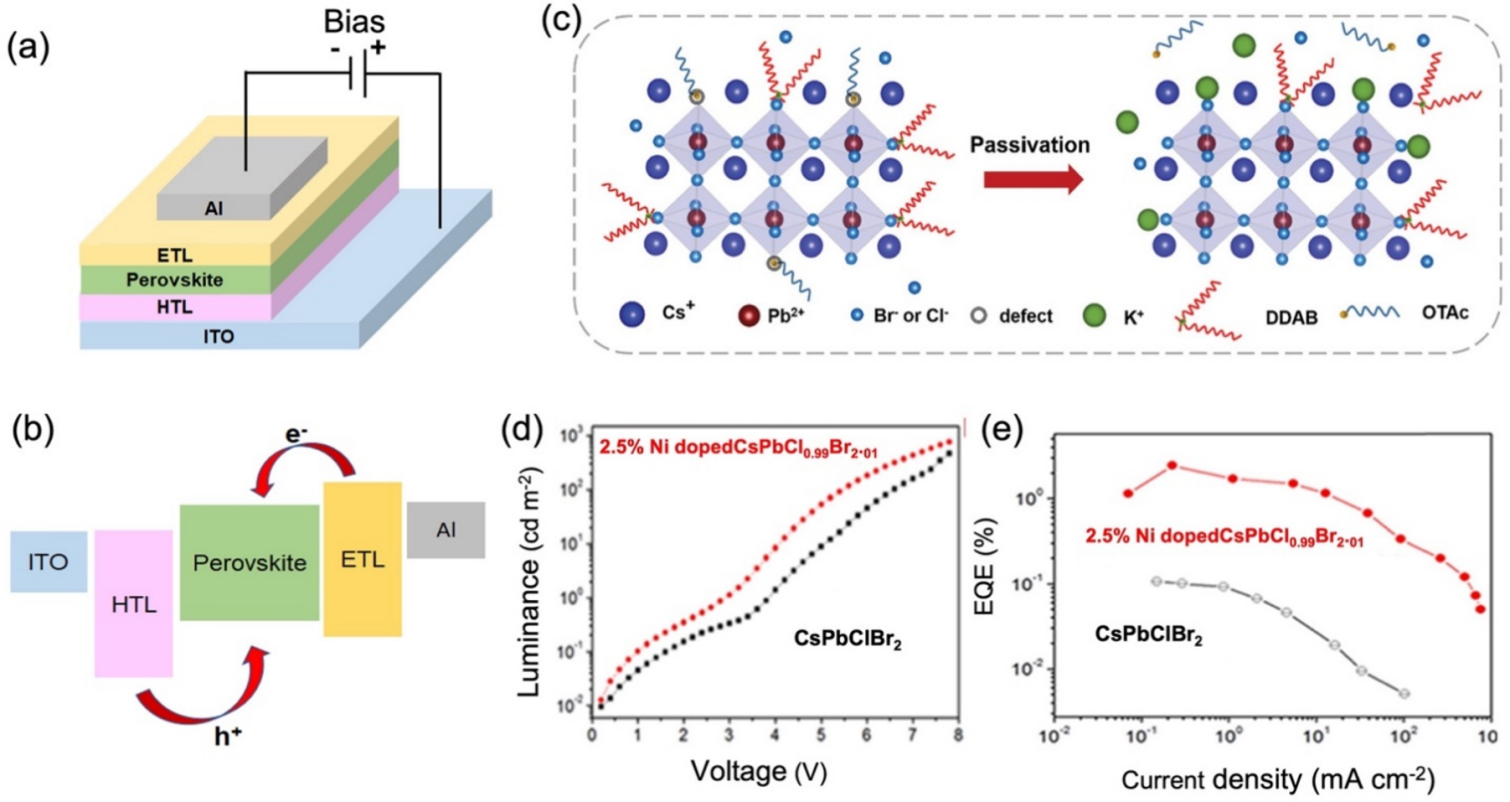
(a) Device structure of a typical perovskite LED. (b) Energy level diagram of a perovskite LED. (c) Schematic illustration of potassium passivation in CsPb(Br/Cl)_3_ NCs. Adapted with permission from ref (12a). Copyright 2020 Wiley. (d) Curve of the luminance as a function of the driving voltage. (e) Curve of the external quantum efficiency of the device as a function of current density for Ni^2+^ ion-doped CsPbCl_*x*_Br_3–*x*_ quantum dots. d, e Adapted with permission from ref (12b). Copyright 2020 American Chemical Society.

As stated before, the quantum efficiency and operational stability of blue emitters have impeded the development of perovskite NC-based white LEDs. The research community is striving to achieve the goal of fabricating stable and efficient blue LEDs. The device architecture (Figure 2a), energy level alignment (Figure 2b), and working principle remain the same for all PLEDs. The high PLQYs in these LEDs can be achieved by suppressing nonradiative recombination pathways. Some promising ways to mitigate nonradiative recombination pathways include metal doping, passivation, and encapsulation.^11a,11b^ Metal doping is primarily achieved by partially replacing Pb^2+^ with other metals at the B-site. Defect sites at the surfaces of NCs are passivated by treating perovskite NCs with ligands. The commonly used ligands (OA and OAm) interact weakly with the perovskite NCs, and they are easily washed off during purification, creating surface traps. OA and OAm also create an insulating barrier around perovskite NCs, impeding the charge transfer and affecting the overall efficiency of devices.^11c^ The pure blue emission is produced by mixed halide (Br/Cl) and pure Br perovskites. However, mixed halide perovskites carry with them the intrinsic ion migration under the application of an external electric field, which is responsible for the segregation of mixed perovskites into Br-rich and Cl-rich regions, therefore causing a shift in the electroluminescence (EL).^11d^ The phase separation has been minimized in quasi-2D perovskites by introducing bulky spacer cations.^11e^

Yang et al. synthesized blue-emitting CsPb(Br/Cl)_3_ NCs by doping them with K^+^ ions (Figure 2c). The LED showed a maximum EQE of ∼1.19% when the [K]/[Pb] concentration was around 4% and a maximum luminance of 399.20 cd/m^2^ at [K]/[Pb] concentrations ∼8%. This emission increase is attributed to the reduction in nonradiative recombination, while the higher current density is attributed to the improved conductivity of perovskite films achieved by improvement in the surface morphology due to a rational amount of K^+^.^12a^ With the introduction of Ni^2+^, the emission of CsPbCl_*x*_Br_3–*x*_ NCs can be tuned from green (508 nm) to blue (432 nm). The halide vacancy, which is responsible for the nonradiative recombination, and exciton trap states were suppressed with Ni^2+^ doping. The highest PLQY of ∼89% at 470 nm emission was achieved in a 2.5% Ni^2+^-doped CsPbCl_0.99_Br_2.01_. The LED constructed with this perovskite NC stoichiometry showed a maximum luminance of 612 cd/m^2^ and a maximum EQE of 2.4%, which is 20× higher than the EQE of the CsPbClBr_2_-based LED, as shown in Figure 2d and e. This optimization in device performance is caused by the valence band modulation, which in turn improved the carrier injection.^12b^

**Figure 3:**
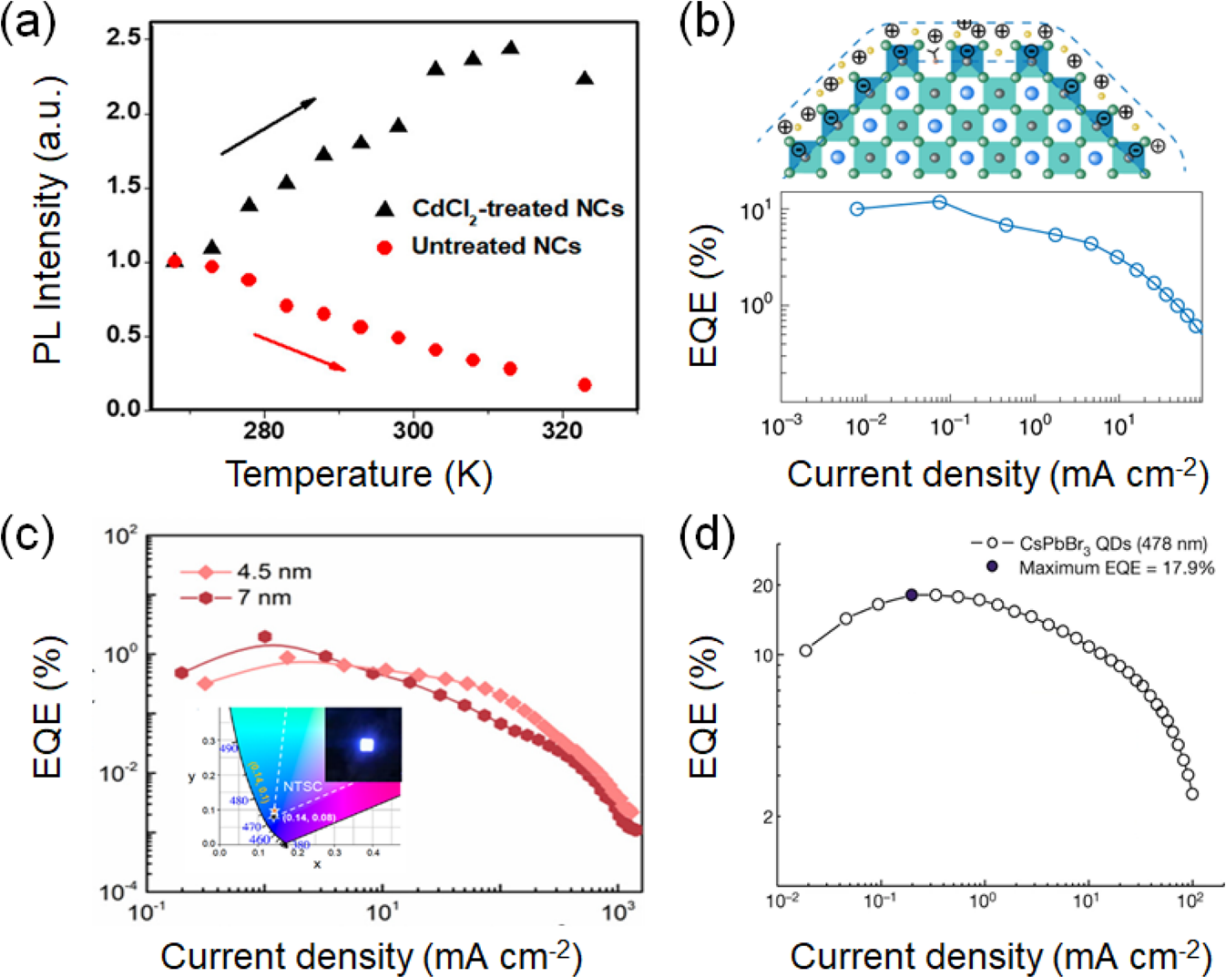
(a) Variation of the PL lifetime of untreated and CdCl_2_-treated CsPbCl_3_ nanocrystals with the temperature (268–328 K). Adapted with permission from ref (12c). Copyright 2019 American Chemical Society. (b) Schematic of the bipolar shell resurfacing of perovskite QDs comprised of an inner anion shell and an outer shell made up of cations and solvent molecules (top). EQE of exchanged blue LEDs with the variation in the current density based on CsPbBr_3_ QDs (bottom). Adapted with permission from ref (14b). Copyright 2020 Springer Nature. (c) EQE–*J* curves of the LED devices based on quantum-confined CsPbBr_3_ nanoplatelet-based 4.5 and 7 nm thick emitter layers. The inset shows the CIE color coordinate and photograph of a LED device (based on a 4.5 nm thick emitter layer) with an emitting size of 4 mm^2^ at an applied current density of 52 mA cm^–2^. Adapted with permission from ref (16a). Copyright 2022 American Chemical Society. (d) Evolution of the EQE with the driving current density for CsPbBr_3_ QDs. Adapted with permission from ref (18a). Copyright 2022 Springer Nature.

Similarly, the doping of CsPbCl_3_ with Cd^2+^ resulted in negative thermal quenching and a longer PL lifetime at higher temperatures (Figure 3a). These intriguing phenomena can be explained by the collision of trap states with excitonic states. The trapped carriers return to the excitonic states and then undergo radiative recombination. CdCl_2_-treated NCs achieved a near-unity PLQY and enhanced air stability for up to 60 days.^12c^ Hou et al. doped the CsPb(Br/Cl)_3_ NCs with Mn^+^ to enhance the PLQY and EQE up to 28% and 2.12%, respectively, at moderate doping. The Mn^+^ dopants neutralize nonradiative centers, which are also manifested by a reduction in Urbach energy values to 14.7 meV.^12d^ Ultrasmall CsPbBr_3_ NCs (∼3 nm) exhibited strong quantum confinement of excitons in ultrasmall dimensions and achieved PLQEs as high as 68% in the blue region. The highly efficient blue emission originates from the radiative recombination of excitons localized in radiative band tail states.^12e^

The halide vacancies can also be reduced by etching perovskite NCs with HBr. The HBr introduced during the synthesis of QDs etches imperfect [PbBr_6_]^4–^ octahedrons, thus removing the surface defects and excess carboxylate ligands from QDs. Thereafter, amine-based ligands were added to bond to the residual uncoordinated sites and facilitate the ligand exchange process. The resulting CsPbBr_3_ QDs possess very low densities of traps with a near-unity PLQY (97%). The LED fabricated using the HBr-treated films with the ITO/PEDOT:PSS/PVK/QDs/ZnO/Ag architecture demonstrated EL at 470 nm, with a maximum EQE of ∼4.7% and a maximum luminance of ∼3850 cd/m^2^_._^13a^ The in situ passivation, which involved the introduction of Br^–^ to neutralize the initial Br vacancies by passivating the uncoordinated Pb^2+^, helped to reduce the nonradiative recombination, leading to the 96% PLQY. The device ITO/PEDOT:PSS/Poly-TPD/CsPbBr_3_ NPLs/TPBi/LiF/Al shows stable EL around 463 nm with a low EQE of ∼0.124%.^13b^ The quantum confinement was also explored in hollow CsPbBr_3_ NCs by manipulating the pore and grain dimensions. The quantum confinement is achieved by introducing Na^+^ and [NH_3_(CH_2_)_2_NH_3_]^2+^ (ethylenediamine, EDA^2+^) during the synthesis process. EDA^2+^ cations partially replaced Pb^2+^ at the B-sites, while Na^+^ occupied interstitial sites. The EDA^2+^ is largely responsible for the passivation of surface defects, and Na^+^ influences the emission color. The highest PLQY of ∼81.5% was obtained at a ratio of 1:1:0 (CsPbBr_3_:EDABr_2_:NaBr).^14a^ Furthermore, bipolar shell resurfacing was proposed to avoid the problem of perovskite decomposition during ligand exchange by polar solvents. The inner shell is composed of anions, and the outer shell is made of cations and polar solvent molecules (Figure 3b). The bipolar shell delays the anion exchange, manifested as a small PL redshift over a longer period. The bipolar shell also inhibits the fast band-edge electron transfer and the increase of the PLQY up to 90% due to the passivation of halide vacancies by the inner shell. The proposed blue LED exhibited enhanced stability (20 min half time at 90 cd/m^2^), improved mobility (0.01 cm^2^/(V s)), a large reduction in trap density, low turn-on voltages, and an EQE of ∼12.3%.^14b^ To improve the stability of CsPbBr_3_ NC-based blue emitters, mesoporous SiO_2_ films were employed as templates.^15a^ Similarly, by embedding CsPbBr_3_ NCs in the Sr^2+^-doped CsPb_1–*x*_Sr_*x*_Br_3_ matrix, LEDs with the device structure ITO/PEDOT:PSS:PFI/QD-in-matrix solid/TPBi/LiF/Al were fabricated. For this configuration to work, the bandgap energy of the perovskite matrix should be higher than that of perovskite NCs to enable efficient charge transport. The LED device containing large NCs inside the matrix showed an impressive EQE of 13.8% with a stable EL maximum at 495 nm.^15b^ Quantum-confined CsPbBr_3_ NPL-based LEDs with an EQE of 2.0% at 463 nm were demonstrated for the first time through the addition of ammonium bromide (NH_4_Br). NH_4_Br controlled the growth of NPLs and simultaneously passivated the defect sites. The postsynthesis treatment of NH_4_Br-treated NPLs with short conjugation ligand–phenethylammonium bromide (PEABr) increases the PLQY from 51.2% to 81.6%. The excess of Br^–^ provided by PEABr also passivated halide vacancies, causing a reduction in nonradiative recombination. The resulting NPLs exhibit excellent spectral stability, with a constant emission peak at variable current densities from 10 to 500 mA/cm^2^. However, 4.5 nm thick NPLs had more intense luminance and a sharper emission peak compared to 7 nm thick NPLs (Figure 3c).^16a^ Interestingly, stable emission over a range of voltages was realized by increasing the activation energy (*E*_a_) for halide ion migration after incorporating FA and GA into CsPbBr_*x*_Cl_3–*x*_ NCs. The FA and GA supply NH_2_ groups that strongly interact with the Pb–X lattice via N–H bonding, which consequently enhanced the PLQY up to 32% and 39% when the NH_2_ sources are GA and FA, respectively. Similarly, the emission features of the corresponding LED are also impacted by the type of NH_2_ source. Luminance also showed an increase from 460 cd/m^2^ for an undoped NC to 603 (GA) and 1762 cd/m^2^ (FA) for doped NCs. Fabricated LEDs exhibit sky blue emission with EQE values reaching 3.02% and 4.14% for Cs/GA and Cs/FA NCs, respectively. The overall improvement was linked to efficient charge injection in doped samples.^16b^

To improve the luminance efficiency and stability of core/shell blue LEDs, there is a need to suppress trap-assisted recombination and optimize the interface hole barrier, which minimizes the efficiency and could cause an imbalance in charge injection, respectively. Park et al. fabricated core/shell CsPbBr_3–*x*_Cl_*x*_/PbBr_*y*_ inverted LEDs to elucidate the effects responsible for luminance roll-off in blue LEDs.^17a^ With the increase in Cl from *x* = 0.57 to *x* = 2.21, the PLQY decreases from 70% to 5.2%, respectively. CsPb(Br_*x*_Cl_1–*x*_)_3_ NCs were passivated with *n*-dodecyl ammonium thiocyanate (DAT) to address this issue. DAT can passivate the Cl^–^ vacancies to avoid electron trapping. Therefore, after passivation, the PLQY increased to 100% at 468 nm, while the resulting LED’s EQE increased to 6.3%.^17b^ Functionalized conjugated polyelectrolytes, such as (poly[(9,9-bis(3′-(*N*,*N*-dimethylamino)propyl)-2,7-fluorene)-*alt*-2,7-(9,9-dioctylfluorene)]) PFN-X, improved charge transport between the CsPbBr_*x*_Cl_3–*x*_ NC active layer and the hole-injecting layer by reducing the hole injection barrier, although the improvement was minimal.^17c^

Ligand engineering was employed to develop ligand structures for the fabrication of a blue LED from ultrasmall monodisperse QDs. CsPbBr_3_ QD formation on the substrate was fulfilled by α-methyl-4-bromide-benzyl-ammonium (Br-MBA^+^). Br-MBA^+^ prevented perovskite layer formation by inducing high octahedral distortion and improved QD formation by regulating the grain size to the quantum confinement regime. The QDs and the corresponding device exhibited the highest PLQY of ∼81% and highest EQE of ∼17.9% at 480 nm, respectively (Figure 3d).^18a^ Overall, these advancements are remarkable and arguably can accelerate the large-scale development of all-inorganic perovskite white LEDs. The overall device architecture, EQE, maximum luminance, and EL peak emission of blue-emitting perovskite NCs discussed in this section are mentioned in Table 2.

**Table 2:** Summary of the Performance of Perovskite NC-Based Blue LEDs

perovskite system	device architecture	EQE (%)	maximum luminance (cd/m^2^)	EL peak (nm)	ref
CsPb(Br/Cl)_3_:K	ITO/PEDOT:PSS/polyTPD/Perovskite/TPBi/LiF/Al	1.19	399.20	476	(12a)
CsPbCl_*x*_Br_3–*x*_:Ni	ITO/PEDOT:PSS/TFB/PFI/Perovskite/TPBi/LiF/Al	2.4	612	470	(12b)
CsPbBr_*x*_Cl_3–*x*_:Mn	ITO/PEDOT:PSS/TFB/PFI/Perovskite/TPBi/LiF/Al	2.12	245	466	(12d)
CsPbBr_3_ QDs	ITO/PEDOT:PSS/PVK/Perovskite/ZnO/Ag	4.7	3850	470	(13a)
CsPbBr_3_ NPLs	ITO/PEDOT:PSS/Poly-TPD/Perovskite/TPBi/LiF/Al	0.124	62	463	(13b)
CsPbBr_3_ QDs	ITO/PEDOT:PSS/PTAA/Perovskite/TPBi/LiF/Al	12.3		470	(14b)
CsPb_1-x_Sr_*x*_Br_3_	ITO/PEDOT:PSS:PFI/Perovskite/TPBi/LiF/Al	13.8	6000	495	(15b)
CsPbBr_3_ NPLs	ITO/PEDOT:PSS/PTAA/PEABr/Perovskite/TPBI/LiF/Al	2.0	74	463	(16a)
(Cs/FA)PbBr_*x*_Cl_3–*x*_ NCs	ITO/PEDOT:PSS/PTAA/Perovskite/TPBi/LiF/Al	4.14	1762	492.5	(16b)
(Cs/GA)PbBr_*x*_Cl_3–*x*_ NCs	ITO/PEDOT:PSS/PTAA/Perovskite/TPBi/LiF/Al	3.02	603	490.5	(16b)
CsPb(Br_*x*_Cl_1–*x*_)_3_ QDs	ITO/poly-TFB/PFI/Perovskite/3TPYMB/Liq/Al	6.3	465	470	(17b)
CsPbBr_*x*_Cl_3–*x*_ NCs	ITO/PEDOT:PSS/poly-TPD/PFN-I/Perovskite/TPBi/LiF/Al	1.34	46.7	470	(17c)
CsPbBr_3_ QDs	ITO/PEDOT:PSS/PVK/Perovskite/TPBi/LiF/Al	17.9		480	(18a)

## All-Inorganic Perovskite White LEDs

**Figure 4:**
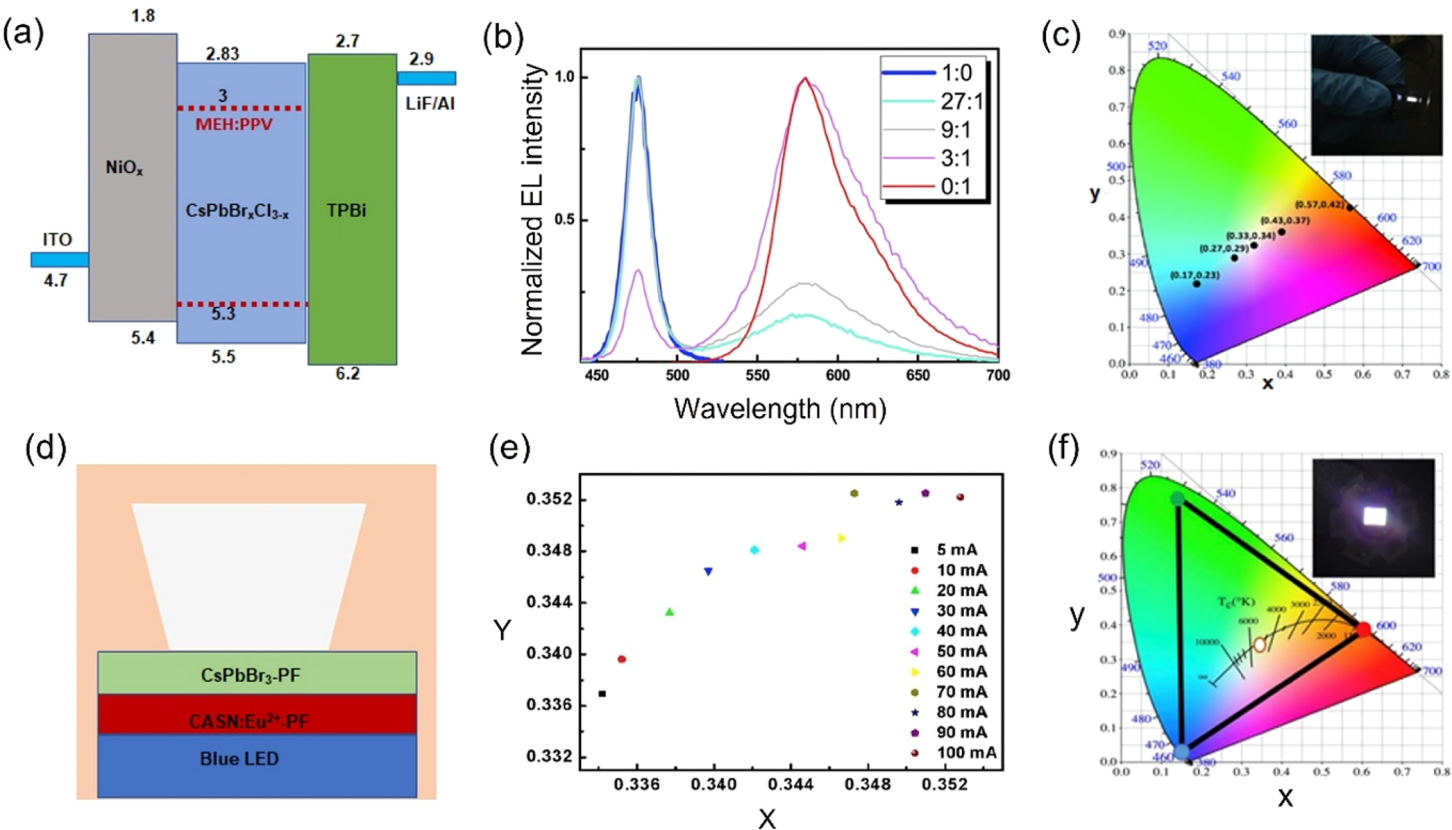
(a) Schematic energy band structure of a CsPbBr_*x*_Cl_3–*x*_ nanocrystal-based blue LED, (b) EL spectra, and (c) CIE coordinates with different weight ratios for the CsPbBr_*x*_Cl_3–*x*_ nanocrystal and MEH:PPV blend-based white LED (the inset shows the white LED photo). Adapted with permission from ref (18c). Copyright 2017 Wiley. (d) Schematic diagram of a white LED based on CsPbBr_3_ and CSAN:Eu^2^ composite films stacked on a blue LED. (e) Evolution of CIE coordinates of a WLED under various operation currents. (f) Color coordinates of a WLED operated at 20 mA after 30 min. The inset depicts the fully operated WLED. Adapted with permission from ref (19a). Copyright 2018 Elsevier.

In terms of performance, PLEDs have made tremendous progress. An early development in all-inorganic perovskite NCs to generate emissions covering multiple wavelengths involved the demonstration of blue, green, and orange LEDs with excellent color purity. All three emission colors have fwhms <30 nm, and LEDs employing all-inorganic perovskites were fabricated using the ITO/PEDOT:PSS/perovskite/TPBi/LiF/Al architecture. The EL spectra show the emission peaks for blue, green, and orange LEDs at 455, 516, and 586 nm, respectively, providing a direction toward developing RGB for white light emission.^2h^ After tuning the emission spectra of NCs to cover the entire visible spectrum, the white emission was demonstrated by coating the highly fluorescent green-emissive CsPbBr_3_ NCs and red phosphors on a blue indium gallium nitride (InGaN) chip. The intensity of the white LEDs (WLEDs) increased with the increase in the forward-bias current from 20 to 60 mA, although with a slight variation in the color rendering index (CRI) from 93.2 to 91.6.^18b^ Yao et al. synthesized blue-emitting CsPbBr_*x*_Cl_3–*x*_ by blending CsPbBr_3_ and CsPbCl_3_ NCs with poly[2-methoxy-5-(2-ethylhexyloxy)-1,4-phenylenevinylene] (MEH:PPV), as shown in Figure 4a. The purity and intensity of the white LEDs were tuned by optimizing the ratio between CsPbBr_*x*_Cl_3–*x*_ and MEH:PPV. The EL intensity increased with the increase in the MEH:PPV weight ratio, and the white light emission occurred at the weight ratio of 9:1 with International Commission on Illumination (CIE) coordinates at (0.33, 0.34) (Figure 4b and c). However, the MEH:PPV materials enhanced exciton quenching by taking excitons from CsPbBr_*x*_Cl_3–*x*_ through the Forester–Dexter transfer process, as indicated by the time-resolved PL.^18c^ By stacking CsPbBr_3_ NCs:ethyl acetate and CaSrAlN_3_:Eu^2+^-poly(methyl methacrylate) composite films on the blue LED chip (Figure 4d), the ideal white light with CIE chromaticity coordinates at (0.3379, 0.3432) and a correlated color temperature (CCT) of 5261 K was obtained. When the operation current was increased from 5 to 100 mA, the chromaticity coordination varied from 0.33 to 0.35 and the CCT changed from 4500 to 5500 K, indicating that the operating current feebly influenced the luminescence performance of the WLED device (Figure 4e and 4f).^19a^

For the first time, an all perovskite WLED was demonstrated with (CH_3_CH_2_CH_2_NH_3_)_2_CsPb_2_I_7_/BIPO:Poly-TPD (1:1)/CsPb(Br, Cl)_3_. The layers are stacked one by one, and the interlayer is designed to simultaneously function as (i) a spacer to isolate PA_2_CsPb_2_I_7_ (PA = CH_3_CH_2_CH_2_NH_3_) and CsPb(Br,Cl)_3_ to eliminate the ion-exchange reaction and (ii) carrier distributors/transporters in the multilayered emission structure. The white color is produced when the red light emitted from (CH_3_CH_2_CH_2_NH_3_)_2_CsPb_2_I_7_ is superimposed onto the cyan light emitted from CsPb(Br/Cl)_3_ NCs. Large energy barriers between the valence band (VB) of PA_2_CsPb_2_I_7_ and the HOMO of BIPO, as well as between the conduction band (CB) of CsPb(Br/Cl)_3_ and the LUMO of Ploy-TPD, are responsible for the steady white emission. In this design, CIE coordinate fluctuation is also very small (0.322 ± 0.002 or 0.316 ± 0.007) depending on the applied voltage.^19b^

**Figure 5:**
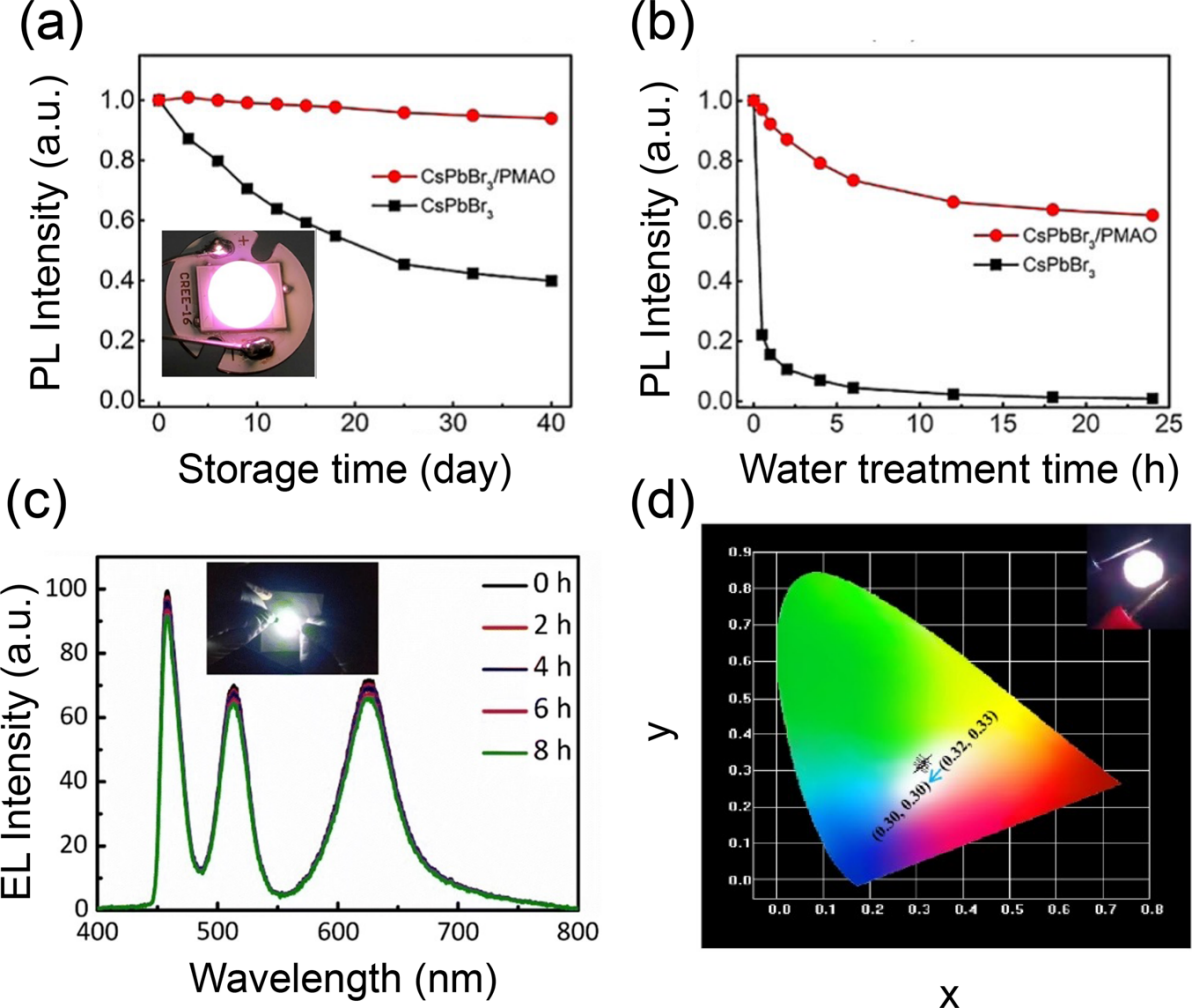
(a) PL stability in air and (b) water resistance of pristine CsPbBr_3_ NCs and PMAO-coated CsPbBr_3_ NCs. The inset in (a) shows the photograph of a working LED. Adapted with permission from ref (19c). Copyright 2019 American Chemical Society. (c) EL spectra under continuous work of the white LED based on a GaN-based blue chip, green-emitting CsPbBr_3_ QDs/PDMS, and a red-emitting CsPbBrI_2_ QDs/PDMS layer operated continuously for 8 h (inset shows a digital photograph of the working white LED). Adapted with permission from ref (21). Copyright 2019 Springer. (d) Corresponding color coordinates of the WLED at different driving currents. The inset shows the working image of the WLED at 5 mA. Adapted with permission from ref (23a). Copyright 2021 Elsevier.

Both air and water stability were improved by coating the CsPbBr_3_ NCs with poly(maleic anhydride-*alt*-1-octadecene) (PMAO) polymers. Warm white emission with CIE coordinates at (0.390,0.332) was realized when PMAO-coated green-emitting CsPbBr_3_ perovskite NCs were used with the blue-emitting InGaN chip and red-emitting nitride phosphor. The PMAO-coated CsPbBr_3_ NCs possess good stability, as demonstrated by the minimal decrease in the PL intensity after exposure to air for 40 days as well the superior water resistance due to the hydrophobic nature of the PMAO polymer. (Figure 5a and b).^19c^ The power efficiency, maximum luminance, CRI, and CCT of WLEDs with PMAO-coated CsPbBr_3_ NCs are mentioned in Table 3.

**Table 3:** Summary of the Performance of Perovskite NC-Based White LEDs

perovskite system	power efficiency (lm/W)	maximum luminance (cd/m^2^)	CIE (*x*, *y*)	CRI (%)	CCT (K)	ref
CsPbBr_3_ QDs			0.33, 0.36	93.2	5447	(18b)
CsPbBr_*x*_Cl_3–*x*_–MEH:PPV		350	0.33, 0.34			(18c)
CsPbBr3 QDs-EC + CSAN:Eu^2+^-EC composite			0.34, 0.34		5261	(19a)
CsPb(Br,Cl)_3_			0.32, 0.32		6000	(19b)
PMAO-coated CsPbBr_3_	56.6	6000000	0.39, 0.33		3320	(19c)
CsPbX_3_-SiO_2_/Al_2_O_3_	80.91		0.37, 0.36	83.8	4082	(20a)
CsPbCl_3_:Bi^3+^/Mn^2+^			0.33, 0.29		4250–1900	(20b)
CsZn_*x*_Pb_1-x_X_3_ NCs	286–318		0.33, 0.36	84–93	2218–8335	(22a)
CsPbBr_2.2_Cl_0.8_: Tm^3+^/Mn^2+^			0.33, 0.34	91		(22b)
CsPbBr_3_@SiO_2_ NCs-CsPbBr_0.6_I_2.4_@SiO_2_	80		0.32, 0.33	90	6000	(23a)
CsPbBr_3_@SiO_2_–AgInZnS QDs	40.6		0.40, 0.41	91	3689	(23b)

To improve the stability of WLEDs in humid air, the red phosphors based on Mn-doped CsPbCl_3_ NCs with a SiO_2_/Al_2_O_3_ monolith (SAM) composite were used to modify the Ce^3+^:YAG-based white LEDs. Mn-doped CsPbCl_3_–SAM phosphors were stacked on a Ce^3+^:YAG phosphorin glass (Ce-PiG) plate using screen-printing technology. The combination of orange-emitting Mn-doped CsPbCl_3_/SAM, yellow-emitting Ce/PiG, and blue-emitting IGaN produces warm white emission. SAM protects NCs from moisture and oxygen attack and imparts stability against blue radiation. The SAM composite-based Mn-doped CsPbCl_3_ also produced an outstanding optical performance with a luminescence efficiency of 80.91 lm/W, a high CRI of 83.8, and a low CT of 4082 K under a 20 mA operational current.^20a^ Furthermore, transition metal doping has evolved as a prominent technique to tailor the electro-optic properties of semiconductors. The dopants can be employed to generate white light emission from a single perovskite composition. Shao et al. observed three transitions from Bi^3+^/Mn^2+^ codoped CsPbCl_3_ perovskite NCs: the blue-emitting component originating from the excitonic transition of the NC host, the green-emitting component associated with the intrinsic transition of Bi^3+^ ions, and the red-emitting band centered at 600 nm associated with the intrinsic transition of Mn^2+^ ions.^20b^ Therefore, benefiting from the dual Bi^3+^ and Mn^2+^ ion dopants, emission with tunable CT from 19000 to 4250 K was achieved by adjusting only the ion doping concentrations. Under the excitation of 365 nm, white light emission with a quantum yield (QY) of 4.2% and CIE color coordinates of (0.33, 0.29) was achieved in the codoped NCs (8.7% doping concentration of Bi^3+^ ions and 2.5% doping concentration of Mn^2+^ ions).^20b^

A stable white LED was fabricated by growing inorganic CsPbX_3_ (X = I or Cl) NCs within the polydimethylsiloxane (PDMS) matrix. Green-emitting CsPbBr_3_ and red-emitting CsPbBrI_2_ NCs were integrated with a GaN-based blue emitter to realize white light emission. The LED displayed stable EL spectra after 8 h of continuous operation (Figure 5c).^21^ To make eco-friendly all-inorganic perovskite-based WLEDs, the alternative for toxic Pb^2+^ also requires attention. To address this challenge, Thapa et al. incorporated Zn^2+^ in the CsZn_*x*_Pb_1–*x*_X_3_ NCs to produce green–blue emissions without compromising the PLQY.^22a^ White light emission was obtained when four different layers of CsZn_*x*_Pb_1–*x*_X_3_, i.e., blue-emitting (CsZn_0.15_Pb_0.85_(Cl_0.5_Br_0.5_)_3_), green-emitting (CsZn_0.15_Pb_0.85_Br_3_), yellow-emitting (CsZn_0.15_Pb_0.85_(I_0.5_Br_0.5_)_3_), and red-emitting (CsZn_0.15_Pb_0.85_(Br_0.25_I_0.75_)_3_), were stacked on a blue-emitting UV LED chip. This configuration helped to produce both warm and cold WLEDs by controlling the ratio of four different stoichiometries. For example, warm white light was realized when yellow- and red-emitting NCs were increased in comparison to blue- and green-emitting NCs.^22a^ Luo et al. proposed a new method to tune the emission spectra by doping CsPbBr_2.2_Cl_0.8_ with Tm^3+^ and Mn^2+^. In addition to improving the exciton energy transfer from the mixed perovskite to Mn^2+^, the change in the concentration of Tm^3+^ induces spectral tunability from green to orange, giving overall white emission.^22b^ By combining core/shell green-emitting CsPbBr_3_/SiO_2_ and red-emitting CsPbBr_0.6_I_2.4_ with a blue GaN chip, a stable WLED displaying stable EL spectra up to 10 h with a minimal variation over the range of driving current was fabricated (Figure 5d).^23a^ In a similar direction, SiO_2_-coated CsPbBr_3_ NCs with a PLQY of ∼75% led to white light emission when used in combination with red AgIZnS and blue IGaN. The resulting LED showed a power efficiency of 40.6 lm/W, a CRI of ∼91, and a CCT of ∼3689 K.^23b^ In summary, perovskite white LEDs represent an emerging area of research that can play a significant role in the energy sector and lighting technology.

## Outlook

Solution-processable white LEDs, a flourishing area of research, can contribute to energy conservation and pollution reduction by replacing conventional and less efficient devices. Having discussed all-inorganic perovskite white lighting technology in this Review, we demonstrated two potential strategies to generate electroluminescent white light using CsPbX_3_ perovskite nanocrystals: (1) by combining multiple LED chips or RGB emitters (multicolor combination) and (2) by employing (co)doped perovskite NCs in a single chip configuration. For the first technology, the three RGB emitters can be combined either vertically or horizontally. A horizontal architecture is preferred to avoid anion exchange. Although in recent reports researchers reported the use of intercalation layers to prevent anion exchange reactions,^21^ three main concerns remain unaddressed: (i) to achieve a uniform white light distribution, the driving currents of different LED chips need to match well with each other; (ii) building a less-complex feedback control system; and (iii) balancing the aging and temperature of individual chips. From the materials perspective, maximizing the operational stability of the blue subpixel (e.g., mixed Cl/Br NCs or quantum-confined nanoplatelets) remains a major concern. LEDs generally need a high driving voltage with currently available contacts. However, the NCs break down under the applied bias required to inject charge carriers for a reasonable electroluminescent output.^24a^ We believe that the instability issues originate from the interface between the inorganic core and the organic capping ligand shell; to make substantial progress, more thoughtful studies are needed to elucidate the impact of this interface on the formation of metallic lead (Pb^0^) and/or on the structural stability of the perovskite lattice. Moreover, heat dissipation strategies could also be crucial for optimizing a robust device design.

For the second technology, the single-chip system would be ideal for white LEDs, as it does not require a complex circuit design. The best example here reported to date is the white EL from Sm^3+^-doped CsPbCl_3_ NCs.^24b^ However, the quality of white light in these systems strongly depends on the dopant concentration, a factor that may vary easily under operational conditions. To maximize the efficiency of this class of emitters, the perovskite host should contain the lowest number of defects, as they significantly quench the EL associated with the dopants. In addition, the (positive or negative) effects of different dopants (e.g., Mn, Cd, Sm, etc.) on the stability of host perovskite NCs when sandwiched in a device stack and under continued operation have not been well studied thus far. The energy transfer from the perovskite host to the dopants should be further investigated, as the carriers can be easily populated on the defect level under the electrical excitation. This event can further introduce additional broad-emitting components in the EL spectra and alter the chromaticity coordinates.

Finally, perovskite-inspired metal halide NCs (e.g., CsCu_2_I_3_) with broadband emission on a single chip can be potentially explored for white lighting. The PL of these types of compounds is mainly based on self-trapped exciton emission with a large Stokes shift. However, there are few reports of EL efficiency from those materials to date. Unfavorable electronic properties, i.e., deep valence-band maxima, low PLQEs, and large effective masses of carriers (leading to poor charge transport), are the main challenges. Therefore, the production of efficient white EL from broadband-emission metal halides remains a challenge. Interestingly, indirect bandgap NCs exhibit strong exciton–phonon coupling, which results in nonradiative self-trapped excitons (STEs), while direct bandgap NCs exhibit moderate exciton–phonon coupling, inducing bright STE PL.^24c^ Therefore, to realize efficient white EL, it is also crucial to develop desired charge injection layer in order to facilitate effective carrier transport and hole injection.
